# Fatigue Effect on Minimal Toe Clearance and Toe Activity during Walking

**DOI:** 10.3390/s22239300

**Published:** 2022-11-29

**Authors:** Yingjie Jin, Yui Sano, Miho Shogenji, Tetsuyou Watanabe

**Affiliations:** 1 Graduated School of Natural Science and Technology, Kanazawa University, Kakuma-machi, Kanazawa 920-1192, Japan; 2 Faculty of Health Sciences, Institute of Medical, Pharmaceutical and Health Sciences, Kanazawa University, Kanazawa 920-0942, Japan; 3 Faculty of Frontier Engineering, Institute of Science and Engineering, Kanazawa University, Kakuma-machi, Kanazawa 920-1192, Japan

**Keywords:** gait, fatigue, forefoot activity, minimal toe clearance

## Abstract

This study investigates the effects of fatigue on the process of walking in young adults using the developed clog-integrated sensor system. The developed sensor can simultaneously measure the forefoot activity (FA) and minimum toe clearance (MTC). The FA was evaluated through the change in the contact area captured by a camera using a method based on a light conductive plate. The MTC was derived from the distance between the bottom surface of the clog and ground obtained using a time of flight (TOF) sensor, and the clog posture was obtained using an acceleration sensor. The induced fatigue was achieved by walking on a treadmill at the fastest walking speed. We evaluated the FA and MTC before and after fatigue in both feet for 14 participants. The effects of fatigue manifested in either the FA or MTC of either foot when the results were evaluated by considering the participants individually, although individual variances in the effects of fatigue were observed. In the dominant foot, a significant increase in either the FA or MTC was observed in 13 of the 14 participants. The mean MTC in the dominant foot increased significantly (*p* = 0.038) when the results were evaluated by considering the participants as a group.

## 1. Introduction

Fatigue is a common experience linked with a lack of physical and mental energy [1,2,3,4]. Fatigue reduces motor performance and can affect an individual’s walk, leading to an increased risk of falling. The effect of fatigue on gait or body balance has been investigated by several researchers. In particular, the influence of fatigue on the gait of older adults has been examined. Santos et al. reported that experimentally induced fatigue affected gait features, including stride length, width, and gait velocity [4]. They examined the effect of age on intermuscular beta band (15–35 Hz) coherence during treadmill walking, before and after experimentally induced fatigue [5]. Granacher et al. examined the effects of muscle fatigue on gait characteristics under single- and dual-task conditions [6]. Recently, considerable attention has been paid to the influence of fatigue on the gait of young adults. Lehnen et al. investigated the effects of triceps surae fatigue and weight training level on gait variability and local stability [7]. Kao et al. investigated the effect of motor fatigue on dual-task walking performance [8]. Coco et al. investigated the effect of exhaustive exercise on posture and gait balance in young adults [9]. The effect of subjective fatigue sensation on gait has also been considered [10,11,12,13]. However, unclear gait features have been associated with fatigue. Examples are the effects on toe activity and minimum toe clearance (MTC).

Furthermore, the importance of foot activity has been recognized [14,15]. During a normal walk, the heel initially makes ground contact. Even a small tilt in the foot during ground contact increases the risk of stumbling. This also makes it difficult to climb stairs. There are several methods for evaluating foot activity. A correlation between ankle dorsiflexion range of motion (DF ROM) and balance has been reported [16]. Another study provides a correlation between the DF ROM and toe flexor strength [17], while the toe flexor strength is reported to be related to the incidence of falls [18,19]. Giandolini et al. investigated the strength of plantar flexors and fatigue in forefoot and rearfoot runners and showed that the pressure maxima decreased under the exposed foot regions after fatigue [20,21]. Urbaczka et al. reported that the ankle angles of insufficiently trained runners changed after an exhaustive running protocol [21]. We evaluated the association between toe activity and trip risk and discovered that, regardless of age, decreased toe activity was related with trip risk [22]. Toe activity was evaluated based on the change in contact area obtained using our sensing clog, which could monitor the visualized plantar aspect [23].

One of the major criteria for evaluating foot activity is the MTC, which is defined as the distance between the toe and ground during the swing phase of gait. A low MTC indicates that the step height that can be climbed without tripping is small. Nagano et al. investigated the effect of fatigue caused by walking on the MTC and reported that the MTC decreased with fatigue in older adults but not in young adults [24]. Watanabe evaluated the variability of MTC during prolonged walks and showed that the MTC decreased significantly with time in older adults but not in young adults [15]. Pereira and Gonçalves investigated the effect of fatigue on motion patterns by walking on a treadmill for 20 min and showed that this fatigue was not high enough to change the motion patterns in older adults [25]. The effect of fatigue on MTC has not been well-investigated [14]. A technique to imitate the fatigue effect in older adults has recently been developed [26]. Mills et al. compared treadmill walking in young and older adults and reported no age-related differences in the median of the MTC. However, the within-subject variability of the MTC was greater in older adults [27], although they did not consider the fatigue effect. The results suggest that the influence of factors such as fatigue and age on gait patterns varies by individual.

**Figure 1:**
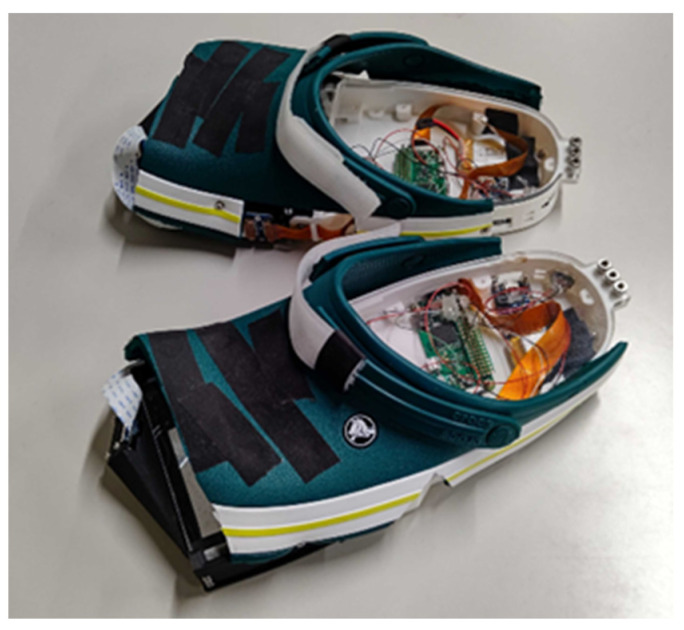
Sensor-integrated clogs for measuring FA and MTC.

This study examines the hypothesis that there are individual differences in the effects of fatigue on gait patterns and that the effect is more likely to appear in the forefoot activity (FA) in some people and in the MTC in others. For this examination, we developed a sensor-integrated clog that can simultaneously measure FA and MTC (Figure 1). The clog developed in this study is used to investigate the impact of fatigue on gait patterns.

Several shoe-integrated walking-measurement systems have been developed [28]. One commonly used sensor is an insole pressure sensor, and some of the recently developed sensors are commercially available [29,30]. Majumder et al. [31] used sensory data from a smartphone and four pressure sensors installed in a shoe to anticipate falls. Ayena et al. [32] used a similar system to estimate the fall risk at home. Yu et al. [33] developed shoe-integrated force sensors. Crea et al. [34] used optoelectronic technology to develop a pressure-sensitive foot insole. Tao et al. developed a smart insole system that monitors plantar pressure in real time using an array of capacitive-pressure sensors with a vertical pore elastic dielectric layer [35].

Another typical method to monitor walking is the Inertial Measurement Unit (IMU) sensor-based system [36]. Hung and Suh [37] used an IR camera to reduce the position error of shoes estimated by IMU sensors. Do and Suh [38] utilized a camera and inertial sensors to estimate the foot angle and step length. Foxlin [39] used shoe-mounted IMU sensors to track pedestrians. Sim et al. [40] used acceleration sensors installed in a shoe to detect falls. Mariani et al. [41] estimated the gait spatial parameters using IMU sensors in a shoe. Rampp et al. [42] assessed a stride parameter using IMU sensors in a shoe.

Insole pressure and IMU sensors can be used simultaneously. Scheoers et al. [43] incorporated force and IMU sensors into a shoe. Bamberg et al. [44] embedded an accelerometer, a gyroscope, and a ground force reaction sensor in a shoe, while Hegde et al. [45] embedded pressure and acceleration sensors in a shoe. Such shoes with multiple sensors have been applied in personal navigation systems [46], anomaly detection [47], activity detection [48], and the detection of gait events [49].

A popular method for monitoring MTC is based on motion capture [50], which requires external vision sensors. Several studies have attempted to estimate the MTC using wearable sensors. Ultrasonic [51,52], time-of-flight (TOF) [53,54,55], and radar [56,57,58] sensors have been used to measure the distance between the ground floor and foot to estimate the MTC.

The sensor-integrated clog developed in this study is an updated version of the model presented in a previous study [53]. The built-in camera measures the plantar aspect directly. The main difference between the existing wearable sensors (especially force or pressure sensors) and sensor-integrated clog is that the clog provides contact area information during walking. A typical difference between the contact pressure and area information is that the contact area information during the swing phase is distinct, while the contact force information during the swing phase is not. Thus, the information obtained from the contact area is not the same as the information obtained from the contact force. This study focuses on the change in the contact area in a single gait cycle and evaluates the change as the FA. It also focuses on MTC as a major gait parameter, as mentioned above. A wearable sensor that can simultaneously monitor the contact area and MTC is required to evaluate both the FA and MTC. The sensor previously developed by us was the first attempt at developing such a system [53].

## 2. Clog-Integrated Sensor System for Measuring FA and MTC

### 2.1. Overview

**Figure 2:**
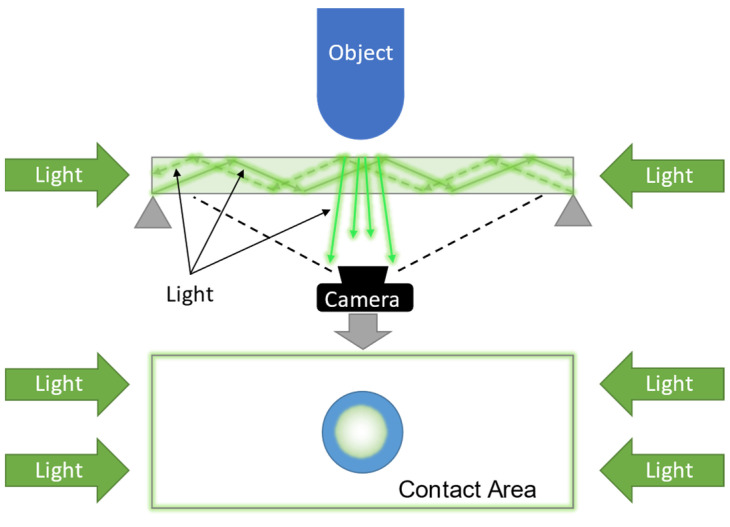
Overview of the contact area deviation method based on the use of a light-conductive plate.

Figure 1 shows the sensor-integrated clog developed for the measurement of the FA and MTC. The built-in camera measures the plantar aspect directly. The MTC can be derived from the distance between the bottom surface of the clog and ground, obtained using a TOF sensor, and the clog posture can be obtained using an acceleration sensor. The main difference between the approach of this study and that of the previous study [53] lies in the strategy used for determining the contact area to derive the FA. A method based on a light-conductive plate [59] was employed in this study. A light-conductive plate, such as a transparent acrylic board, was installed between the object and camera, as shown in Figure 2. The light beam is emitted onto the light-conductive plate (transparent acrylic plate in this study) from the sides. If an object makes contact with the surface of the plate, it reflects the light at the contact point and scatters it, which is captured by a camera. This method has three main advantages over the previous method, as it directly observes the plantar aspect using a camera while walking. First, it is unaffected by ambient light. The previous method used ambient light; thus, the foot posture affected the brightness of the captured image, which decreased the accuracy of the system. Second, it can provide contact area information without any delay. The previous method detected the contact area by detecting the skin area that turned white owing to capillary blood flow obstruction caused by the contact load. As a result, there was a temporal lag in detecting the contact. In contrast, the slight contact scatters the light beam in the method involving the use of a light-conductive plate, allowing the contact to be detected without a time delay. Therefore, its sensitivity and accuracy are higher than those achieved using the previous method, which can be considered the third advantage.

### 2.2. Structure

**Figure 3:**
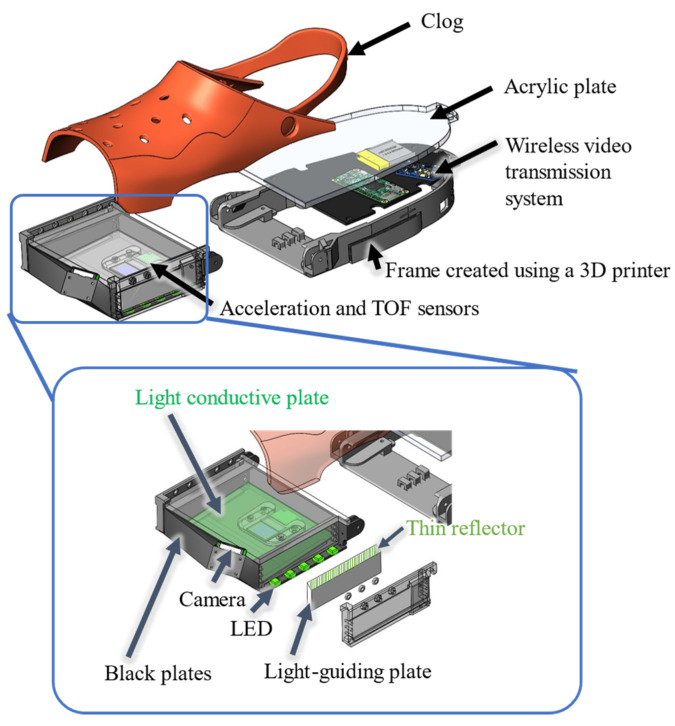
Overview of the clog-integrated sensor system developed for measuring the forefoot contact area and minimum toe clearance.

**Figure 4:**
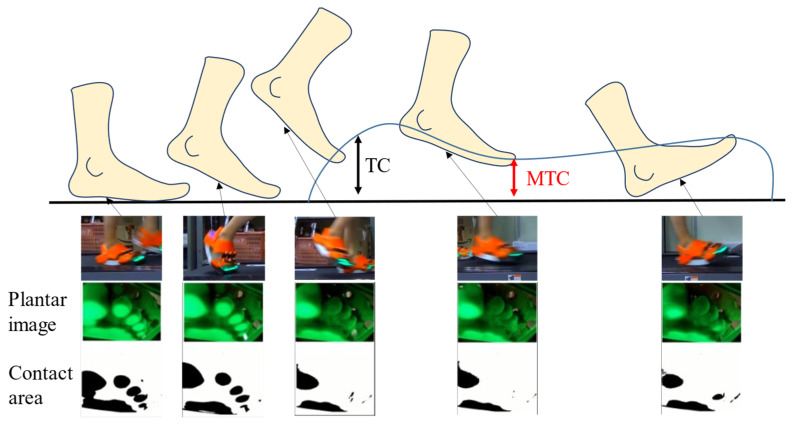
Toe clearance, minimum toe clearance, observed plantar image, and extracted contact area.

Figure 3 shows an overview of the clog-integrated sensor system developed for measuring the forefoot contact area and MTC. The system for measuring the MTC was the same as the sensor system used in the previous study [53]. As shown in Figure 3, acceleration (MPU-6050) and TOF (VL6180X) sensors were installed on the plate corresponding to the forefoot. The TOF sensor provides the distance between the ground and sensor position, while the bottom posture of the clog can be derived from the acceleration of the forefoot of the clog. From the distance and clog posture, the distance between the forefoot and ground—that is, the toe clearance (TC)—can be derived (Figure 4). The minimum TC during the swing phase is the MTC. This procedure is described in detail in our previous study [53]. The forefoot contact area is derived using the method based on the light-conductive plate, which is different from the method used in the previous study [53]. As shown in Figure 3, the LED (SK6812) is embedded in the bottom area, and light from the LED passes through the side plate and is emitted into the light-conductive plate. The side plate is the light-guiding plate, composed of a transparent acrylic plate sandwiched between the black plates to block the light. The tip of the light-guide plate is chamfered at 45°, and a thin reflecting film is attached to the chamfered area, such that the light source can be reflected in the chamfered area and emitted into the light-conductive plate. The light-conductive plate is composed of a transparent acrylic plate to support human weight, and a thin reflecting film is bonded to the acrylic plate to aid light conductivity. If the forefoot makes contact with the light-conductive plate, light from the LED scatters. The scattered light can be captured attaching a camera (Raspberry camera v2, Raspberry Pi, UK) to the front of the clog (Figure 3). This method enables highly sensitive and accurate contact information to be obtained without a time delay or ambient light.

The upper part of the clog Is composed of a commercially available clog (Crocs, Niwot, CO, USA). The frames of the lower part were manufactured using a 3D printer (Raise 3D Pro2) to support human weight and minimize the entire weight of the sensor system. A passive joint was installed between the forefoot and rest of the clog, as shown in Figure 3, to minimize the effects of the solid parts on the process of walking.

### 2.3. Wireless Data Transfer

**Figure 5:**
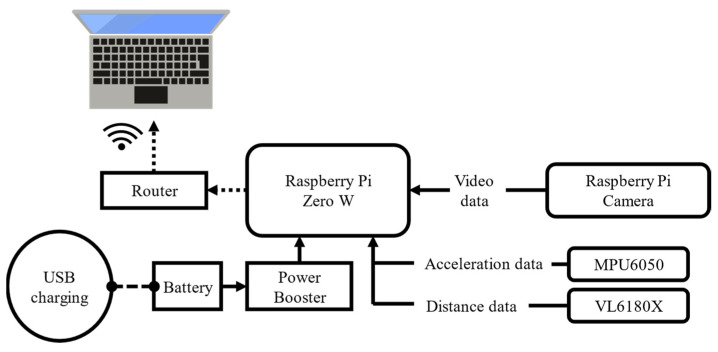
Wireless transmission system.

A wireless data transfer was accomplished by embedding the entire processing system into the clog and using a wireless local area network. Figure 5 shows a schematic illustration of the wireless transfer system. The main device used in the data transfer was a Raspberry Pi Zero W. The data from the acceleration and TOF sensors were input to the general purpose input/output interface of the Raspberry Pi. The Raspberry Pi camera was connected to the Raspberry Pi with a camera serial interface (CSI) cable and dedicated interface connector. The CSI cable was fixed along the edge of the 3D-printed frame and attached to the camera fixed at the tip area of the clog (Figure 1 and Figure 3). A detachable battery module (500-mA·h) and power boost module were used to power the Raspberry Pi and sensors. The video data collected on the Raspberry Pi were transmitted to an external computer via wireless communication using a router. The data from the acceleration and TOF sensors were saved in the storage of the Raspberry Pi and wirelessly transmitted to the external computer. All the devices were embedded in the clog, as shown in Figure 1 and Figure 3.

### 2.4. Deviation of Contact Area and Corresponding FA

**Figure 6:**
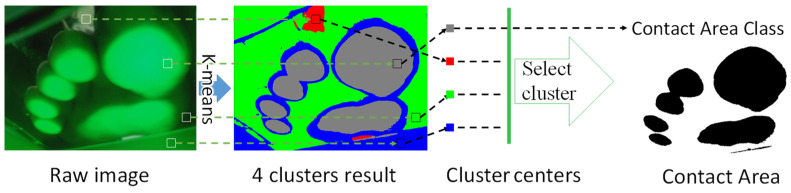
Overview of the contact area extraction process.

**Figure 7:**
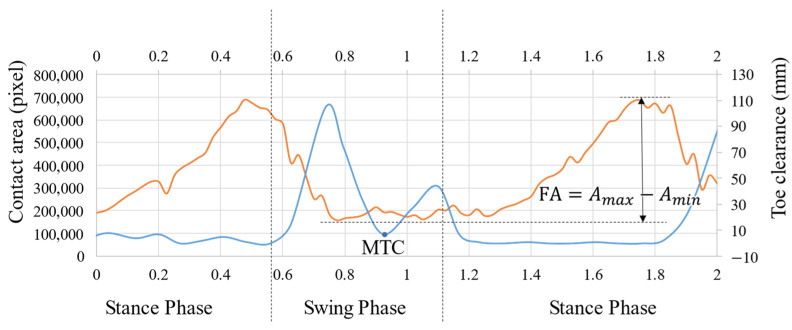
Representative results of the extracted contact area. The orange line represents the contact area, while the blue line represents the TC.

An overview of the contact area extraction process is depicted in Figure 6. The captured image provides information regarding the contact area at the plantar aspect. Even a slight contact of the foot can be detected; however, the image may include noise. Therefore, we performed the following steps. First, the original image was converted to a hue, saturation, and value (HSV) image, and only the value channel was extracted as a feature. Subsequently, k-means clustering was employed, and the number of clusters was determined based on the Calinski–Harabasz Index. As the position of the forefoot does not change significantly during walking, the position of the contact area can be approximated as a region. Among all the clusters, the one corresponding to the contact area was extracted by checking whether the cluster location was within the assumed contact area. Figure 7 shows the representative results of the extracted contact area while walking in this manner. The orange line represents the contact area, while the blue line represents the TC. The contact area is greatest near the end of the stance phase and lowest during the swing phase. The TC exhibits a two-peak waveform during the swing phase, and the minimum value between the two peaks is referred to as the MTC.

Previously [22,23], the change in the contact area during the stance phase was evaluated, because the contact area information during the swing phase was not distinct. In contrast, the updated sensor can monitor the contact area, even during the swing phase. Therefore, we evaluated the FA based on the change in the contact area in a single gait cycle. Here, $A_{max}$ and $A_{min}$ denote the maximum and minimum peaks of the contact area in a single walking cycle, respectively. We evaluated the FA as: $\mathrm{FA}=A_{max}-A_{min}$.

## 3. Materials and Methods

### 3.1. Participants

There were 14 participants in the study, including seven men and seven women (age 24.3 ± 1.8 years, weight = 57.5 ± 10 kg, height = 165.8 ± 8.2 cm, BMI = 20.9 ± 3.6 kg/m^2^, and foot size = 25 ± 1.8 cm) without any disease, impediment, or medical history involving ambulatory problems. The dominant foot of all the participants was the right foot. This study was approved by the Medical Ethics Committee of Kanazawa University (No. 33). All the procedures were conducted in accordance with the ethical standards of the institutional and national research committee and the 1964 Declaration of Helsinki, its later amendments, and comparable ethical standards. After receiving a complete explanation about the study, all the participants agreed to take part and provided written informed consent.

### 3.2. Procedure

**Figure 8:**
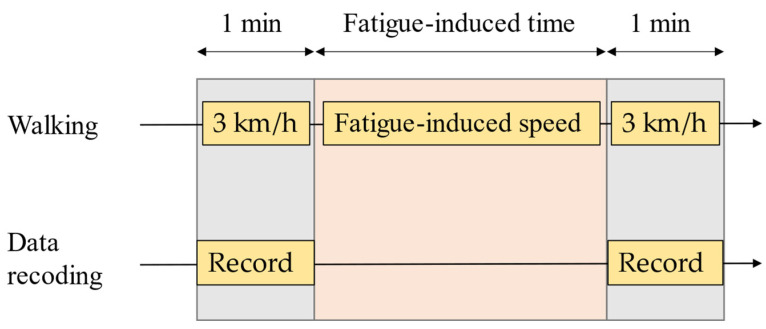
Procedure of the main experiment.

The experiment consisted of a preparatory experiment and main experiment. In the preparatory experiment, an explanation of the overall experiment; a description of the study, content details, and precautions were provided to the participants; and written informed consent was obtained from them. Subsequently, the participants wore easy-to-move shoes and walked on the treadmill at the fastest walking speed, which was accepted as the fatigue rate of the individual (we call this speed the fatigue-induced speed). The Borg rating of perceived exertion (RPE) 14-point scale (6 as “no exertion at all” to 20 as “maximal exertion”) was used as the reference for the fatigue rate. The participants walked at the fatigue-induced speed until the RPE 15 point (“hard–heavy”) was attained, and the total walking time of the process was recorded as the fatigue-induced time. Finally, to familiarize themselves, the participants wore the developed sensor clogs on both feet, adjusted them, and practiced walking to reduce the possibility of accidents. In the main experiment (Figure 8), the participant first walked for 1 min with sensor clogs on both feet at a speed of 3 km/h; plantar images and TC were recorded during walking. Next, the participant changed into easy-to-move, comfortable shoes and walked on the treadmill for the fatigue-induced time at the fatigue-induced speed determined in the preparatory experiment. Finally, they walked for 1 min wearing the sensor clogs on both feet at a speed of 3 km/h again to record the plantar image and TC.

### 3.3. Data Analysis

**Figure 9:**
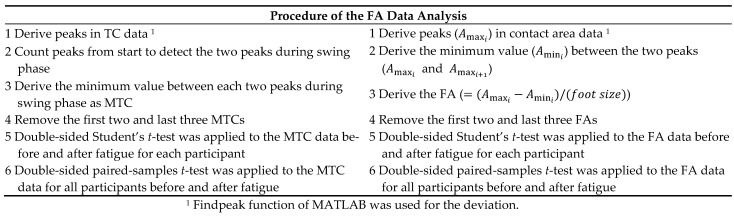
Procedure of the data analysis.

We compared the FA and MTC for each participant before and after walking fatigue. MATLAB (MathWorks) was used to perform the statistical analyses. Walking data for the first two and last three cycles were excluded, and the FA and MTC were evaluated for the remaining walking cycles during the 1-min walk. Figure 9 depicts the procedure for the data analysis. In the analysis of the MTC data, we first derived the peaks in the TC data during the 1-min walk using the Findpeak function of MATLAB. Subsequently, the two peaks during the swing phase were detected by counting the peaks from the beginning. The minimum value between each two peaks was derived as the MTC. After removing the first two and last three values in the MTC data vector, a double-sided Student’s *t*-test was applied to the MTC data before and after fatigue. Similarly, we first derived the peaks ($A_{\max_{i}},\;i\in \left\{1,2,\cdots \right\}$) in the contact area data using the Findpeak function and determined the minimum value ($A_{\min_{i}}$) between the two peaks ($A_{\max_{i}}$ and $A_{\max_{i+1}}$). Subsequently, the FA ($=(A_{\max_{i}}-A_{\min_{i}})/\left(foot\;size\right)$) was derived and normalized using the foot size that was obtained from the interview. After removing the first two and last three values in the FA data vector, a double-sided Student’s *t*-test was applied to the FA data before and after fatigue for each participant. We also applied a double-sided paired-samples *t*-test to the MTC and FA data for all participants before and after fatigue. The significance level in the analysis was set at 0.05.

## 4. Results

### 4.1. Evaluation of Fatigue Effects by Considering All the Participants as a Group

**Figure 10:**
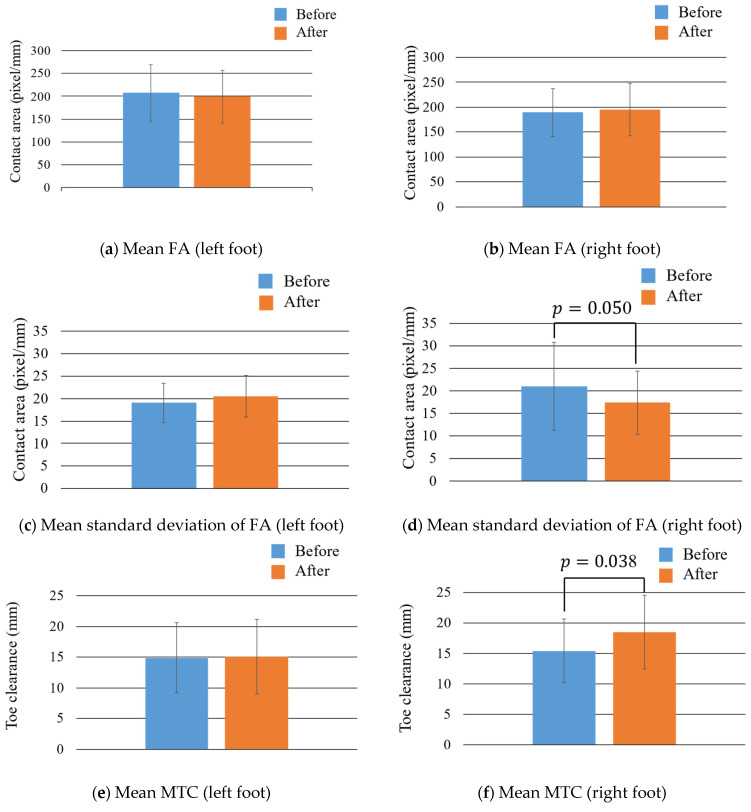

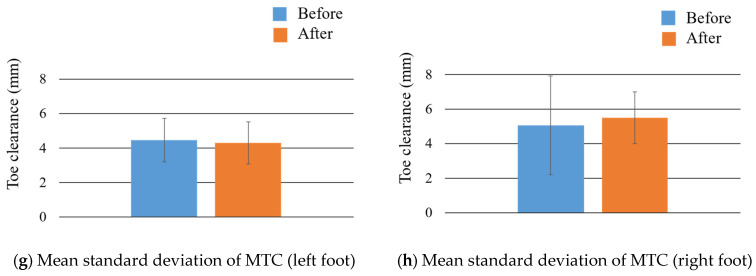
Mean values and mean standard deviation values of FA and MTC for all the participants.

Figure 10 and Table 1 show the mean and mean standard deviation values of the FA and MTC for all the participants and the changes in them owing to fatigue. Upon comparing the results before and after fatigue, it was observed that the mean value for MTC of the right foot (*p* = 0.038) increased significantly. The mean standard deviation value of FA decreased in the right foot (*p* = 0.050), although not significantly. The rate of change was calculated as follows: (FA or MTC value after fatigue)/(FA or MTC value before fatigue), where a value greater than 1 indicated an increase and a value less than 1 indicated a decrease.

### 4.2. Evaluation of Fatigue Effects by Considering the Participants Individually

**Figure 11:**
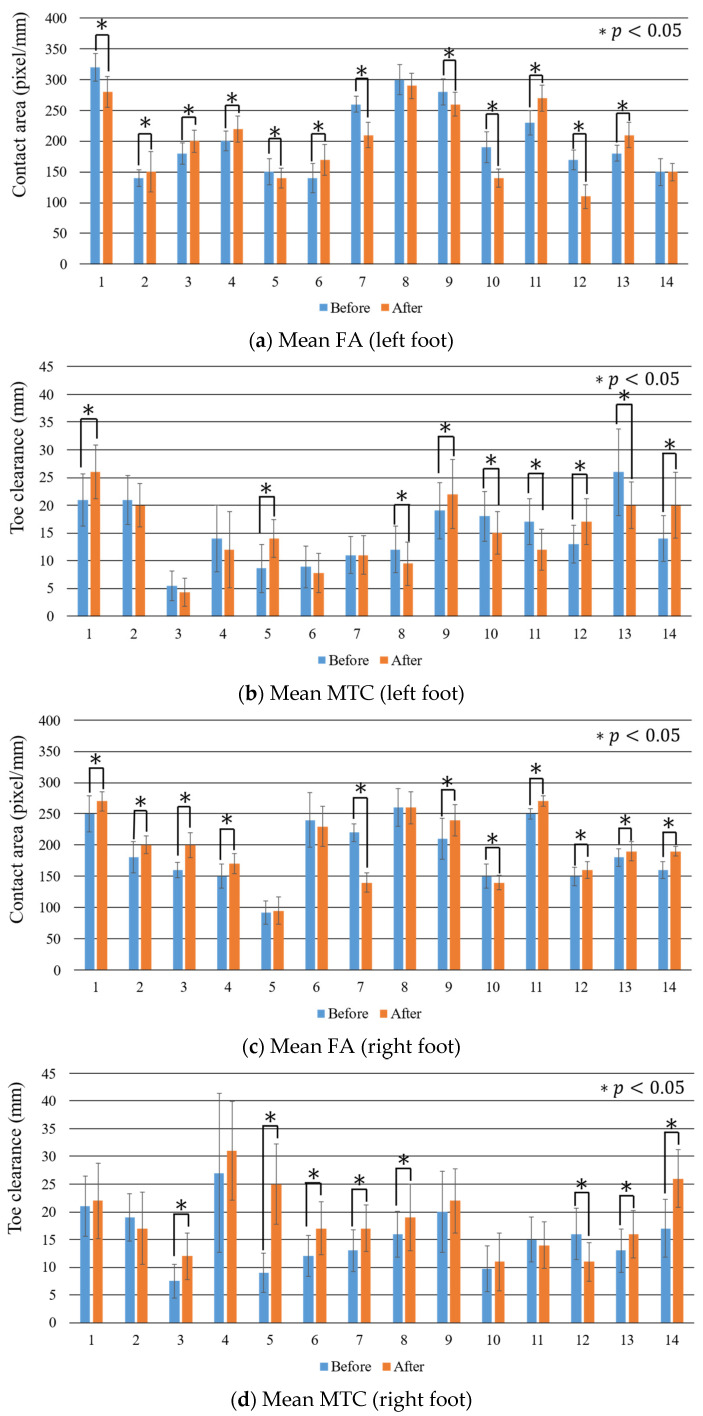
Mean FA and MTC of the individual participants.

Figure 11 and Table 2 show the mean FA and MTC for the individual participants and the changes in them owing to fatigue. We counted the number of participants who showed a significant increase or decrease in the FA or MTC values owing to fatigue. Table 3 summarizes the results.

In the left foot (Table 3a), a significant increase in the FA was observed in six participants, a significant decrease in the FA was observed in six participants, a significant increase in the MTC was observed in five participants, and a significant decrease in the MTC was observed in four participants.

In the right foot (Table 3b), a significant increase in the FA was observed in nine participants, a significant decrease in the FA was observed in two participants, a significant increase in the MTC was observed in seven participants, and a significant decrease in the MTC was observed in one participant.

There were no participants with significant differences in either the FA or MTC owing to fatigue. A significant increase in either the FA or the MTC was observed in 11 of the 14 participants in the left foot and in 13 of the 14 participants in the right foot.

Combinations of the types of changes in the FA and MTC were examined. There were two combinations that were observed most frequently (4 of the 14 participants) in the left foot: (1) the combination in which the FA increased significantly and the MTC did not change significantly and (2) the combination in which the FA decreased significantly and the MTC increased significantly. The most frequent combination in the right foot was the one in which the FA increased significantly and the MTC did not change significantly (5 of the 14 participants).

## 5. Discussion

### 5.1. Evaluation of Fatigue Effects by Considering All the Participants as a Group

As mentioned in Section 4.1, the MTC of the right foot significantly increased owing to fatigue. The fatigue considerably decreased the variability in the FA in the right foot, although the decrease was insignificant. The right foot was the dominant foot in all the participants. The compensation of the effects of fatigue is attributed to the changes in the gait strategy of the dominant foot.

The gait strategy of increasing MTC in the right foot is considered a compensatory action. Chiba et al. reported that, upon comparing the MTC of older adults who had fall experience (the experience of falling while walking) with older adults who did not have fall experience, they found that the MTC decreased in the older adults who had fall experience [60]. No clear decrease in the MTC was observed as a group, and it could be attributed to the fact that the participants in this study were young adults. It is considered that the young participants perform a compensatory action to increase the MTC, considering that fatigue can decrease the MTC and foot height in the swing phase. Mills et al. reported that a large variability in the MTC indicated a high risk of stumbling [28]. No distinct change in the MTC variability was observed as a group, although an increase in the MTC variability was observed in 11 of the 14 participants individually (Table 2 and Figure 11). The compensatory action of lifting the foot minimized the increase in the risk of tripping caused by fatigue (increase in MTC).

Another gait strategy for mitigating the effects of fatigue is to reduce the variability in the FA in the dominant (right) foot. In the walking motion, the leg that swings forward (the leg in the swing phase) is responsible for transferring the body weight to the supporting leg (the leg in the stance phase), and the supporting leg is responsible for forward propulsion [61]. The shift in the center of mass when the dominant leg is raised (while the nondominant leg is the supporting leg) to start walking is greater than when the nondominant leg is raised to start walking [62]. Thus, the nondominant leg is mainly used for supporting the body weight and forward propulsion, whereas the dominant leg is mainly used for fine walking motion. Here, we refer to this as the common usage of the dominant/nondominant leg during walking. Thus, it is presumed that fatigue makes it difficult to adopt a strategy to finely control the walking motion by changing the FA. This loss of control may be the reason for the increase in MTC variability in 11 of the 14 participants.

### 5.2. Evaluation of Fatigue Effects by Considering the Participants Individually

Due to fatigue, none of the participants had a constant FA or MTC in either foot. This indicates that the effects of fatigue appear in either the FA or MTC of either foot.

The number of participants whose FA or MTC changed significantly was approximately the same for the left and right feet. In contrast, the number of participants with significantly increased FA or MTC on the dominant (right) foot was greater than the number of participants with increased FA or MTC on the nondominant (left) foot. In the nondominant (left) foot, individual differences were observed in the manifestation of fatigue effects.

In the dominant (right) foot, a significant increase in the FA or MTC was observed in 13 of the 14 participants (the FA significantly decreased in the remaining participant). Considering the common usage of the dominant/nondominant leg during walking, as mentioned earlier, it is presumed that the change in the load on the supporting foot due to the transfer of the bodyweight and forward propulsion when the nondominant foot is the supporting foot is greater than when the dominant foot is the supporting foot. This could be the reason for the FA in the left (nondominant) foot being greater than that in the right foot before fatigue. The difference in the FA between the left and right feet decreased after fatigue. It was presumed that fatigue made it difficult to adopt the common usage of the dominant/nondominant leg during walking and that the gait strategy wherein the body weight is supported by both legs was adopted. This can be the reason for the increase in the FA in the dominant (right) foot in a high percentage of participants (9 out of 14). The reason for the increase in the MTC in the dominant (right) foot is presumed to be the same as that mentioned in Section 5.1, namely, a compensatory action to mitigate an increase in the risk of stumbling.

Next, the relationship between the changes in the FA and MTC was considered. The most frequent combination in both feet was the one in which the FA increased significantly, and the MTC did not change significantly. Since fatigue made it difficult to raise the foot as needed, the strategy of increasing the maximum contact area of the foot (associated with pressure) was employed to raise the foot as it was before fatigue. Another frequent combination in the left foot was the one in which the FA decreased significantly and the MTC increased significantly (4 out of 14 participants). The FA in the right foot increased significantly or did not change in the four participants. It was presumed that the four participants adopted the strategy of supporting the body weight with both feet (as in the case of increased FA in the right foot) while raising the left leg higher than before fatigue to compensate for the decrease in the FA in the left foot.

In summary, two gait strategies were adopted. One involved supporting the body weight with both feet, and the other involved raising the leg higher than it was before fatigue. The participants employed various combinations of these two strategies in their post-fatigue gait strategy.

From the interviews, it was observed that participants 1, 5, and 11 experienced tripping. Among them, participants 1 and 5 walked more than one hour every day. It might be one of the reasons why the MTC in participant 11 decreased in both feet, while the MTC in other participants who exercised on a daily basis increased in both feet (raising the leg higher than it was before fatigue). However, the reason was not well-founded, because all the participants who exercised on a daily basis did not always have increased MTC in either foot. Participants 2, 4, 10, and 14 also walked for more than one hour every day. Participant 13 had 1.5 h of muscle training per week. Four of the seven participants who exercised on a daily basis had increased MTC in either foot. Five of the seven participants who exercised on a daily basis had increased FA in the dominant foot, which could be associated with the gait strategy of supporting the body weight with both feet. However, a convincing association was not found between the fatigue effects on the MTC and FA and tripping experiences or daily exercise routines.

### 5.3. Limitation: Future Lines of Research

The participants wore the newly developed clog-integrated sensor system in the experiments; thus, the results of this study cannot be generalized to cases where other footwear is used. The target participants were healthy young adults, and the results could be different for other age groups and individuals with diseases. Further studies could investigate the effects of fatigue in other age groups and in people with diseases. Induced fatigue was limited to physical fatigue, and other types of fatigue, such as mental fatigue, can be further investigated.

## 6. Conclusions

In this study, we investigated the effects of fatigue on the process of walking in young adults using a clog-integrated sensor system. The effect of fatigue on gait or body balance has been investigated, but the influence of fatigue on the gait of young adults remains unclear. Examples of unclear gait features associated with fatigue are the effects on forefoot activity (FA) and minimum toe clearance (MTC). We focused on the FA associated with the variance in contact area during walking and investigated the effects of fatigue on the FA and MTC. The contact area information is different from the contact pressure information and thus cannot be obtained using conventional insole pressure sensors. Therefore, we developed a new clog-integrated sensor system that can simultaneously measure the FA and MTC. The system is an updated version of the previous system developed by us, and the main difference lies in the installation of a light-conductive plate to measure the contact area of the plantar aspect to derive the FA. The installation allowed the contact area of the swing phase to be clearly defined and the change in the contact area during a single gait to be evaluated. Other benefits of this new approach include being unaffected by ambient light, the absence of information delay, and improved sensitivity and accuracy. With this updated sensor, we evaluated the FA and MTC before and after fatigue in both feet for the 14 participants. Induced fatigue was achieved by walking on a treadmill at the fastest walking speed. The results indicated that the effects of fatigue appeared in either the FA or MTC of either foot, although there were individual differences. The effects were different for the dominant and nondominant feet. The number of participants with significantly increased FA or MTC in the dominant foot was greater than the number of participants with increased FA or MTC in the nondominant foot. In the dominant foot, a significant increase in either the FA or MTC was observed in 13 of the 14 participants. In the nondominant foot, individual differences were observed in the manifestation of fatigue effects. When the results were evaluated as a group, the mean MTC in the dominant foot increased significantly. The fatigue also decreased the mean standard deviation of the FA in the dominant foot, although not significantly. Based on the results, the participants adopted two gait strategies: supporting the body weight with both feet and raising the leg higher than it was before fatigue. The participants employed various combinations of these two strategies in their post-fatigue gait strategy.

The results presented may be applied to estimating the effect of fatigue on walking when a user has different exercise opportunities, such as travel and sightseeing, in their daily life. The results can also serve as a warning to the user when walking is affected by fatigue and the risk of tripping or falling increases.

## Figures and Tables

**Table 1:** Mean values and mean standard deviation values of FA and MTC for all the participants and changes in them owing to fatigue.

	Before	After	Rate of Change	*p*-Value ^1^
Left foot				
FA (mean (pixel/mm) ± std.)	210 ± 62	200 ± 58	0.95	0.41
FA (mean of std.)	19 ± 4.3	21 ± 4.6	1.1	0.45
MTC (mean (mm) ± std.)	15 ± 5.7	15 ± 6.1	1.0	0.88
MTC (mean of std.)	4.5 ± 1.2	4.3 ± 1.2	0.96	0.67
Right foot				
FA (mean (pixel/mm) ± std.)	190 ± 49	200 ± 53	1.1	0.47
FA (mean of std.)	21 ± 9.8	17 ± 7.0	0.81	**0.050 ^2^**
MTC (mean (mm) ± std.)	15 ± 5.2	19 ± 6.1	1.3	**0.038 (<0.05) ^2^**
MTC (mean of std.)	5.1 ± 2.8	5.5 ± 1.5	1.1	0.47

^1^*p* is probability value. ^2^ The bold indicates *p* ≤0.05.

**Table 2:** Mean FA and MTC of the individual participant and changes in them owing to fatigue.

(**a**) Left foot				
	Before	After	Rate of change	*p*-Value ^1^
1				
FA (mean (pixel/mm) ± std.)	320 ± 22	280 ± 25	0.88	**0.000** **^2^**
MTC (mean (mm) ± std.)	21 ± 4.7	26 ± 4.9	1.2	**0.000** **^2^**
2				
FA (mean (pixel/mm) ± std.)	140 ± 13	150 ± 33	1.1	**0.022** **^2^**
MTC (mean (mm) ± std.)	21 ± 4.4	20 ± 3.9	0.95	0.77
3				
FA (mean (pixel/mm) ± std.)	180 ± 17	200 ± 18	1.1	**0.000** **^2^**
MTC (mean (mm) ± std.)	5.5 ± 2.7	4.3 ± 2.5	0.78	0.27
4				
FA (mean (pixel/mm) ± std.)	200 ± 16	220 ± 21	1.1	**0.000** **^2^**
MTC (mean (mm) ± std.)	14 ± 6.0	12 ± 6.9	0.86	0.19
5				
FA (mean (pixel/mm) ± std.)	150 ± 21	140 ± 16	0.93	**0.001** **^2^**
MTC (mean (mm) ± std.)	8.6 ± 4.3	14 ± 3.4	1.6	**0.000** **^2^**
6				
FA (mean (pixel/mm) ± std.)	140 ± 24	170 ± 25	1.2	**0.000** **^2^**
MTC (mean (mm) ± std.)	8.9 ± 3.8	7.8 ± 3.5	0.88	0.16
7				
FA (mean (pixel/mm) ± std.)	260 ± 13	210 ± 20	0.81	**0.000** **^2^**
MTC (mean (mm) ± std.)	11 ± 3.3	11 ± 3.5	1.0	0.62
8				
FA (mean (pixel/mm) ± std.)	300 ± 24	290 ± 21	0.97	0.10
MTC (mean (mm) ± std.)	12 ± 4.2	9.5 ± 3.9	0.79	**0.0053** **^2^**
9				
FA (mean (pixel/mm) ± std.)	280 ± 21	260 ± 19	0.93	**0.000** **^2^**
MTC (mean (mm) ± std.)	19 ± 5.1	22 ± 6.2	1.2	**0.040** **^2^**
10				
FA (mean (pixel/mm) ± std.)	190 ± 25	140 ± 15	0.74	**0.000** **^2^**
MTC (mean (mm) ± std.)	18 ± 4.5	15 ± 3.8	0.83	**0.0011** **^2^**
11				
FA (mean (pixel/mm) ± std.)	230 ± 20	270 ± 21	1.2	**0.000** **^2^**
MTC (mean (mm) ± std.)	17 ± 4.1	12 ± 3.7	0.71	**0.000** **^2^**
12				
FA (mean (pixel/mm) ± std.)	170 ± 16	110 ± 19	0.65	**0.000** **^2^**
MTC (mean (mm) ± std.)	13 ± 3.4	17 ± 4.1	1.30	**0.000** **^2^**
13				
FA (mean (pixel/mm) ± std.)	180 ± 13	210 ± 20	1.2	**0.000** **^2^**
MTC (mean (mm) ± std.)	26 ± 7.8	20 ± 4.2	0.77	**0.019** **^2^**
14				
FA (mean (pixel/mm) ± std.)	150 ± 22	150 ± 14	1.0	0.52
MTC (mean (mm) ± std.)	14 ± 4.1	20 ± 5.9	1.4	**0.0022** **^2^**
(**b**) Right foot				
	Before	After	Rate of change	*p*-Value ^1^
1				
FA (mean (pixel/mm) ± std.)	250 ± 29	270 ± 16	1.1	**0.0032** **^2^**
MTC (mean (mm) ± std.)	21 ± 5.4	22 ± 6.8	1.0	0.30
2				
FA (mean (pixel/mm) ± std.)	180 ± 25	200 ± 14	1.1	**0.000** **^2^**
MTC (mean (mm) ± std.)	19 ± 4.2	17 ± 6.5	0.89	0.19
3				
FA (mean (pixel/mm) ± std.)	160 ± 12	190 ± 20	1.2	**0.000** **^2^**
MTC (mean (mm) ± std.)	7.5 ± 3.0	12 ± 4.2	1.6	**0.000**
4				
FA (mean (pixel/mm) ± std.)	150 ± 19	170 ± 16	1.1	**0.000** **^2^**
MTC (mean (mm) ± std.)	27 ± 14	31 ± 8.9	1.1	0.20
5				
FA (mean (pixel/mm) ± std.)	92 ± 19	95 ± 22	1.0	0.55
MTC (mean (mm) ± std.)	9.0 ± 3.6	25 ± 7.3	2.8	**0.000** **^2^**
6				
FA (mean (pixel/mm) ± std.)	240 ± 44	230 ± 32	0.96	0.23
MTC (mean (mm) ± std.)	12 ± 3.7	17 ± 4.8	1.4	**0.000** **^2^**
7				
FA (mean (pixel/mm) ± std.)	220 ± 14	140 ± 16	0.64	**0.000** **^2^**
MTC (mean (mm) ± std.)	13 ± 3.8	17 ± 4.2	1.3	**0.000** **^2^**
8				
FA (mean (pixel/mm) ± std.)	260 ± 30	260 ± 26	1.0	0.98
MTC (mean (mm) ± std.)	16 ± 4.1	19 ± 6.0	1.2	**0.012** **^2^**
9				
FA (mean (pixel/mm) ± std.)	210 ± 33	240 ± 25	1.1	**0.000** **^2^**
MTC (mean (mm) ± std.)	20 ± 7.3	22 ± 5.8	1.1	0.15
10				
FA (mean (pixel/mm) ± std.)	150 ± 19	140 ± 11	0.93	**0.000** **^2^**
MTC (mean (mm) ± std.)	9.7 ± 4.1	11 ± 5.2	1.1	0.32
11				
FA (mean (pixel/mm) ± std.)	250 ± 8.6	270 ± 8.4	1.1	**0.000** **^2^**
MTC (mean (mm) ± std.)	15 ± 4.0	14 ± 4.2	0.93	0.063
12				
FA (mean (pixel/mm) ± std.)	150 ± 15	160 ± 14	1.1	**0.0054** **^2^**
MTC (mean (mm) ± std.)	16 ± 4.6	11 ± 3.5	0.69	**0.000** **^2^**
13				
FA (mean (pixel/mm) ± std.)	180 ± 14	190 ± 15	1.1	**0.021** **^2^**
MTC (mean (mm) ± std.)	13 ± 3.9	16 ± 4.3	1.2	**0.040** **^2^**
14				
FA (mean (pixel/mm) ± std.)	160 ± 14	190 ± 7.3	1.2	**0.000** **^2^**
MTC (mean (mm) ± std.)	17 ± 5.2	26 ± 5.2	1.5	**0.000** **^2^**

^1^*p* is probability value. ^2^ The bold indicates *p* <0.05.

**Table 3:** Number of participants with a significant increase or decrease in the FA or MTC owing to fatigue (Up indicates a significant increase; Down indicates a significant decrease).

(**a**) Left foot					
		MTC			Subtotal (FA)
		Up	Down	-	
FA	Up	0	2	4	6
	Down	4	1	1	6
	-	1	1	0	2
Subtotal (MTC)		5	4	5	
(**b**) Right foot					
		MTC			Subtotal (FA)
		Up	Down	-	
FA	Up	3	1	5	9
	Down	1	0	1	2
	-	3	0	0	3
Subtotal (MTC)		7	1	6	

## Data Availability

The data presented in this study can be made available upon request from the corresponding author.
